# Causality in Discrete Time Physics Derived from Maupertuis Reduced Action Principle

**DOI:** 10.3390/e23091212

**Published:** 2021-09-14

**Authors:** Roland Riek, Atanu Chatterjee

**Affiliations:** 1 Laboratory of Physical Chemistry, ETH Zurich, CH 8093 Zurich, Switzerland; 2 Department of Physics of Complex Systems, Weizmann Institute of Science, Rehovot 7610001, Israel; atanu.chatterjee@weizmann.ac.il

**Keywords:** causality, Maupertuis principle, discrete time, reduced action

## Abstract

Causality describes the process and consequences from an action: a cause has an effect. Causality is preserved in classical physics as well as in special and general theories of relativity. Surprisingly, causality as a relationship between the cause and its effect is in neither of these theories considered a law or a principle. Its existence in physics has even been challenged by prominent opponents in part due to the time symmetric nature of the physical laws. With the use of the reduced action and the least action principle of Maupertuis along with a discrete dynamical time physics yielding an arrow of time, causality is defined as the partial spatial derivative of the reduced action and as such is position- and momentum-dependent and requests the presence of space. With this definition the system evolves from one step to the next without the need of time, while (discrete) time can be reconstructed.

## 1. Introduction

Causality is the relationship between a cause and its effect [1]. Causality therefore describes the process and consequences from an action. While omnipresent in classical physics [2] as well as special and general theories of relativity [3,4], it is not considered a law or a principle, nor described by a formula. Furthermore, the proposition of causality to be a physical principle is challenged by many philosophers and physicists [5,6,7,8], thus denying its applicability or its usefulness in physics. The schools around Mach and Russell and their followers challenge causality in physics due to several arguments which have been summarized by Frisch [9]:

(i) *Vagueness challenge*: The notions of cause and effect are inherently vague in contradistinction to the mathematical precision characteristic of theories in physics.
(ii) *Dominant cause challenge*: Causal notions can, if at all, only be legitimately employed in contexts in which we can isolate a small set of factors of interest as those responsible for the occurrence of an event—the dominant cause or causes—by drawing a distinction between causes and background conditions. Yet such a distinction, it is argued, cannot be drawn in physics.
(iii) *Determinism challenge*: Causes necessitate their effects, but the fundamental laws of physics are nondeterministic.
(iv) *Locality challenge*: Causal relations are relations among spatio-temporally localized events, yet fundamental physical laws relate entire global time-slices.
(v) *Time-asymmetry challenge*: The notion of cause is generally taken to be temporally asymmetric: effects never precede their causes. Yet, it is usually argued that the dynamical laws of the fundamental or established theories of physics are time-symmetric and have the same character in both temporal directions.

The problem on the existence of causality within physics theory is further illustrated here by citing Poincaré (using an English translation [10] elaborating on the least action principle (see also Equations (1) and (2) below).

“The very enunciation of the principle of least action is objectionable. To move from one point to another, a material molecule, acted upon by no force, but compelled to move on a surface, will take its path along the geodesic line—i.e., the shortest path. This molecule seems to know the point to which we want to take it, to foresee the time that it will take it to reach it by such a path, and then to know how to choose the most convenient path. The enunciation of the principle presents it to us, so to speak, as a living and free entity. It is clear that it would be better to replace it by a less objectionable enunciation, one in which, as philosophers would say, final effects do not seem to be substituted for acting causes [10]”.

In our advent to introduce causality as a physical law and principle, the points stated above need to be taken seriously. In order to do so, causality will be described here by a mathematical precise formulation (challenge (i)), which is deterministic (challenge (iii)) and independent of time (resolving Poincaré’s quest) yielding a chain of causal events that yield a discrete time and as such guarantees time asymmetry (challenge (v)). By doing so, causality is more fundamental than time as also expressed by Reichenbach with the wording that “time order is reducible to causal order” [11]. Challenges (ii) and (iv) will not be considered. The formulation is based on Maupertuis least action principle of classical mechanics (and as such we stay here within mechanics). The Maupertuis reduced action [12,13,14,15] is introduced in Section 2.1 in detail, followed by a discrete time physics theory in Section 2.2 expanded to special relativity in Section 2.3 and discussed in paragraph three.

## 2. Theory

### 2.1. From the Least Action Principle to the Principle of Maupertuis

Following Landau and Lifschitz [12] (for the entire Section 2.1) the least action principle states that the variation:

(1) δS=0

and the action,

(2) $$S\left(q,\dot{q}\right)=\int_{t_{1}}^{t_{2}}L\left(q,\dot{q}\right)dt$$

with the Lagrangian $L(q,\dot{q})$ and the action variation δS expressed by independent variations along the space coordinate components $\delta q_{i}$ on a true path from the defined starting value $q_{i}\left(t_{1}\right)$ to many possible end values $q_{i}\left(t_{2}\right)$ with a defined time point $t_{2}$. The least action principle states that the path with the smallest action is taken, which means the path with the overall smallest sum (i.e., integral) of the Lagrangian *L*. Since, the Lagrangian is the difference between kinetic energy *T* and the potential energy *V* this means that on the path between time point $t_{1}\to t_{2}$ the path is taken which has overall the smallest kinetic energy, or in other words the system likes to follow a path with a maximal potential (which when applied to humans we would call the most “lazy” path). In this description the action *S* is time dependent.

For a system with one degree of freedom this reads:

(3) $$0=\delta S=\frac{\partial S}{\partial q}\delta q+\frac{\partial S}{\partial \dot{q}}\delta \dot{q}=\delta q\frac{\partial L}{\partial \dot{q}}{|}_{t_{1}}^{t_{2}}+\int_{t_{1}}^{t_{2}}\left(\frac{\partial L}{\partial q}-\frac{d}{dt}\frac{\partial L}{\partial \dot{q}}\right)\delta qdt$$

Thus, yielding Newton’s second law $\left(\frac{\partial L}{\partial q_{i}}-\frac{d}{dt}\frac{\partial L}{\partial \dot{q_{i}}}\right)=0$ from the second term expanded to general degrees of freedom because the variations $\delta q_{i}$ at the two time points $t_{1}$ and $t_{2}$ are requested to be 0 because the coordinates at the start and end of the path are defined (i.e., lack of variation). Hence, with the least action principle the motion of the mechanical system, including the trajectory as well as the time points passing through the individual points of the trajectory, is determined.

Next, we change the point of view and study the evolution of the system with the action S(q,t) from a defined starting point $q_{i}\left(t_{1}\right)$ to a fixed end point localisation $q_{i}\left(t\right)$ but with a variation in time *t*. In addition, the system is assumed to be conservative with E=H(q,p)=constant. Hence, the variation of the action is given by,

(4) $$\delta S=\sum \frac{\partial S}{\partial q_{i}}\delta q_{i}+\frac{\partial S}{\partial t}\delta t=\frac{\partial S}{\partial t}\delta t$$

To get insights into the partial time derivative of the action, the definition of the action from Equation (2) and its total time derivative is substituted such that,

(5) $$\frac{dS}{dt}=L=\frac{\partial S}{\partial t}+\sum_{i}\frac{\partial S}{\partial q_{i}}{\dot{q}}_{i}=\frac{\partial S}{\partial t}+\sum_{i}p_{i}{\dot{q}}_{i}$$

The above equation further reduces to the following expression,

(6) $$\frac{\partial S}{\partial t}=L-\sum_{i}p_{i}{\dot{q}}_{i}=-H$$

with *H* being the Hamiltonian of the system under study. This yields,

(7) $$\delta S=\frac{\partial S}{\partial t}\delta t=-H\delta t=-E\delta t$$

On the other hand with,

(8) $$dS=\frac{\partial S}{\partial t}dt+\sum_{i}\frac{\partial S}{\partial q_{i}}dq_{i}=-Hdt+\sum_{i}p_{i}dq_{i}$$

we obtain,

(9) $$S=\sum_{i}\int p_{i}dq_{i}-\int Hdt=\sum_{i}\int p_{i}dq_{i}-E\left(t-t_{1}\right)$$

which yields for the action variation:

(10) $$\delta S=\sum_{i}\delta \left(\int p_{i}dq_{i}\right)-E\delta t$$

which requests because of Equation (7),

(11) $$\sum_{i}\delta \left(\int p_{i}dq_{i}\right)=\delta S_{0}=0$$

We define the reduced action, $S_{0}=\sum_{i}\int p_{i}dq_{i}$, and the Maupertuis least action principle as, $\delta S_{0}=0$. The reduced action has a minimum for all possible trajectories that follow paths of constant energy at arbitrary time points. In other words, time does not play a role. Furthermore, the path between $q_{i}\left(t_{1}\right)\to q_{i}\left(t_{2}\right)$ is taken with the smallest sum (i.e., integral) of the momentum, which is equivalent to the smallest kinetic energy (under the assumption that the momenta keep their sign). Consider, momentum $p_{i}=\frac{\partial L(q,\dot{q})}{\partial \dot{q_{i}}}$ and the total energy $E=E(q,\dot{q})$, and assuming the Lagrangian to be $L\left(q,\dot{q}\right)=\frac{1}{2}\sum_{i}m{\dot{q_{i}}}^{2}-V\left(q\right)$ if restricted to Cartesian coordinates for simplicity, therefore yields,

(12) $$p_{i}=m\dot{q_{i}}\;\mathrm{and}\;E=\frac{1}{2}\sum_{i}m{\dot{q_{i}}}^{2}+V\left(q\right)$$

Together with $\frac{dq_{i}}{dt}=\dot{q_{i}}$ one can solve for time as,

(13) $$dt=\sum_{i}\sqrt{\frac{m\;\;}{2(E-V(q))}}dq_{i}$$

It is interesting to note that time is reconstructed by Equation (13) and depends on the energy of the system and the acting potential in addition to the mass and the space change. Furthermore, in a situation with the entire energy of the system is in the acting potential (i.e., lack of kinetic energy) both the denominator and the numerator are 0, thereby eventually yielding an undefined time step to be studied. Thus, time appears not to be fundamental.

### 2.2. Discrete Time Physics

Let us consider a system that evolves in discrete dynamic steps [16,17,18].

$$\left[q\left(1\right),p\left(1\right)\right],\left[q{\left(2\right)}_{,}p\left(2\right)\right],.....\left[q\left(n\right),p\left(n\right)\right],\left[q\left(n+1\right),p\left(n+1\right)\right],......$$

The evolution of this system is given by the abbreviated action under the condition of minimality,

(14) $$S_{0}=\int \;\sum_{i}p_{i}\;dq_{i}\;\mathrm{with}\;\delta S_{0}=0$$

We define causality connecting the cause at point *n* with the event caused at point n+1 by,

(15) $$C_{i}\left(n\to n+1\right):=\frac{\partial S_{0}\left(q\left(n\right),q\left(n+1\right)\right)}{\partial q_{i}}=\frac{\partial }{\partial q_{i}}\int_{n}^{n+1}p_{i}dq_{i}=p_{i}\left(n+1\right)-p_{i}\left(n\right)$$

because $p_{i}\left(n+1\right)-p_{i}\left(n\right)=\frac{\partial }{\partial q_{i}}\int_{0(p=0)}^{n+1}p_{i}dq_{i}-\frac{\partial }{\partial q_{i}}\int_{0(p=0)}^{n}p_{i}dq_{i}=\frac{\partial }{\partial q_{i}}\int_{0(p=0)}^{n+1}p_{i}dq_{i}+\frac{\partial }{\partial q_{i}}$ $\int_{n}^{0}p_{i}dq_{i}=\frac{\partial }{\partial q_{i}}\int_{n}^{n+1}p_{i}dq_{i}$

Thus, yielding the following expression,

(16) p(n+1)=p(n)+C(n→n+1)

Hence, causality is the change of the abbreviated action along the space coordinate as the system evolves. In other words, causality is the smallest change in the momentum possible for the system to reach the next point. This makes much sense because something that moves with a constant velocity causes nothing (also because the reference frame can be moved with the system of interest). Furthermore, the notion of causality only makes sense if the system is out of equilibrium and thus is subjected to a spatio-temporal heterogeneity due to thermodynamic forces and fluxes, thus resulting in a change in temperature (i.e., change in velocity) and/or an acting potential is required for “action” to happen. At equilibrium, for example, it is important to note that there is no time variable [19,20,21]. Therefore, it is about events that first take place in space, thus resulting in a causal relationship from it to the next step that is achieved. From Equation (13), time can be reconstructed as follows,

(17) $$▵t_{i}\left(n\right)=\int_{n}^{n+1}\sqrt{\frac{m\;}{2(E-V(q))}}dq_{i}$$

Thus, time is being a dynamical discrete variable [16], while space is continuous. Using a discrete form of Newton’s second law, $\frac{p_{i}\left(n+1\right)-p_{i}\left(n+1\right)}{▵t_{i}}=-\frac{\partial V(q(n))}{\partial q_{i}}$ [18] and Equation (17), causality can also be written as,

(18) $$C_{i}\left(n\to n+1\right)=-▵t_{i}\left(n\right)\frac{\partial V(q(n))}{\partial q_{i}}=-\int_{n}^{n+1}\sqrt{\frac{m\;dq_{i}\;dq_{i}}{2(E-V(q))}}\frac{\partial V(q(n))}{\partial q_{i}}$$

which again is free of the variable time. We recently demonstrated that if time is reintroduced as a metric of causality it is required to be of a discrete nature because only with a discrete time step can the cause that yields an event be distinguished [18]. This finding is reinforced here when time is made infinitely small, because the following equation is obtained,

(19) $$\lim_{▵t\to dt}C_{i}\left(n\to n+1\right)=-\sqrt{\frac{m\;}{2(E-V(q(n)))}}\frac{\partial V(q(n))}{\partial q_{i}\left(n\right)}dq_{i}\left(n\right)$$

which is meaningless since there is no $q_{n+1}$ dependency and thus no event is caused by the cause. From a time picture point of view, causality can further be regarded as the product between the time step and acting force ($C_{i}\left(n\to n+1\right)=-▵t_{i}\left(n\right)\frac{\partial V(q(n))}{\partial q_{i}}=F_{i}\left(n\right)▵t_{i}\left(n\right)$), which complements the other process entity “work” being the product between force and the space step (i.e., $F_{i}\left(n\right)dq_{i}\left(n\right)$). Furthermore, from the Hamilton–Jacobi equation [12] the total energy for a conservative system can be written as,

(20) $$H(q_{i},C_{i})=E$$

### 2.3. Expansion to Special Relativity

In this paragraph, we attempt to elaborate on the nature of causality in the special theory of relativity [13] starting with the continuous time case. Both the action principle and reduced action principle can be expanded to special relativity [12,13] with the covariant action *I* (using the notation by Gray) given by,

(21) $$I=\int_{\tau_{1}}^{\tau_{2}}Ld\tau$$

where $\tau =\gamma^{-1}t$ is the proper time (“Eigenzeit”) with $\gamma =\frac{1}{\sqrt{1-{\left(v/c\right)}^{2}}}$. The proper time represents the time in the reference frame that moves with the velocity *v* with the object under study and is located at the object, while time *t* is the time the observer measures and *c* is the velocity of light. It is noted that there are two different descriptions of the action given in the literature, varying by the sign. We follow the notation in [13] which allows for orthodox definitions of the components of the four dimensional Minkowski space time (in Einstein summation notation) with μ=0,1,2,3 and the Minkowski metric (−,+,+,+) of the momentum $p^{\mu }=\frac{\partial L}{\partial v_{\mu }}$ and the Hamiltonian $H=p_{\mu }v^{\mu }-L$. According to [13] the reduced covariant action is,

(22) $$I_{0}=\int_{x_{A}}^{x_{B}}p_{\mu }dx^{\mu }$$

For practical purposes the three dimensional noncovariant action (with μ=1...3) and proper time τ is generally used [13]. In the determination of the proper time, which is covariant since $s^{2}={\left(ct\right)}^{2}-x^{2}={\left(c\tau \right)}^{2}$, for an energy conservative system with $E\;=\;H\;=\;V\left(x\right)+m_{0}c^{2}$ (with $m_{0}$ the rest mass) in the moving reference frame a dτ step later $E=V\left(x+\mathrm{dx}\right)+mc^{2}=V\left(x+\mathrm{dx}\right)+m_{0}c^{2}/\sqrt{(1-{\left(\frac{dx}{d\tau }/c\right)}^{2}}$. The time step dτ is then given by,

(23) $$\begin{array}{c} d\tau =\sum_{i}{\left(\frac{dx_{i}dx_{i}}{c^{2}\left(1-{\left(\frac{m_{0}c^{2}}{E-V(x)}\right)}^{2}\right)}\right)}^{1/2}=\sum_{i}{\left(\frac{dx_{i}dx_{i}}{c^{2}\left(1-{\left(\frac{E-V(x)}{E-V(x+\mathrm{dx})}\right)}^{2}\right)}\right)}^{1/2}\\ =\sum_{i}{\left(\frac{dx_{i}dx_{i}}{c^{2}\left(1-{\left(\frac{m_{0}}{m}\right)}^{2}\right)}\right)}^{1/2} \end{array}$$

If a second order approximation of the energy is evaluated, $E=V\left(x+\mathrm{dx}\right)+mc^{2}\approx V\left(x+\mathrm{dx}\right)+m_{0}c^{2}+\frac{1}{2}m_{0}{\left(\frac{dx}{d\tau }\right)}^{2}$ then,

(24) $$d\tau \approx \sum_{i}\sqrt{\frac{m_{0}\;dx_{i}\;dx_{i}}{2(V(x+dx)-V(x))}}$$

approximating the classical case.

To expand the findings to discrete time physics, it is noted, that the discrete nature of time is not mixed with the continuous nature of space because (assuming for simplicity a one dimensional system), ${\left(v/c\right)}^{2}={\left(\left(x_{n+1}-x_{n}\right)/\Delta \tau \right)}^{2}/{\left(\left(x_{n+1}^{c}-x_{n}^{c}\right)/\Delta \tau \right)}^{2}={\left(\left(x_{n+1}-x_{n}\right)/\left(x_{n+1}^{c}-x_{n}^{c}\right)\right)}^{2}$ with $x_{n}^{c}$ the wave front coordinate vector of light at time point *n*, and the Lorentz transformation holds as demonstrated in [22]. With the reduced action $I_{0}$ causality is given by,

(25) $$C_{i=1..3}\left(n\to n+1\right)=\frac{\partial I_{0}}{\partial x_{i}}$$

in analogy to the classical case as shown in Equation (18). To calculate the proper time step for an energy conservative system with $E=H=V\left(x_{n}\right)+m_{0}c^{2}$ in the moving reference frame a ▵τ(n) step later $E=V\left(x_{n+1}\right)+mc^{2}$ and the proper time as the covariant metric of causality can be determined by (please note, $m=m_{0}\gamma$ is independent of the time step),

(26) $$▵\tau_{i}\left(n\right)=\sqrt{\frac{\left(m_{0}-m\right){\left(x_{i,n+1}^{c}-x_{i,n}^{c}\right)}^{2}}{V\left(x_{n+1}\right)-V\left(x_{n}\right)}}$$

## 3. Discussion

In the present approach a definition of causality within mechanics and special relativity is provided which relies on the least action principle of the reduced action, also called the Maupertuis principle. It is demonstrated that mechanical causality can be defined as the partial derivative of the reduced action along the space coordinate with the request on the smallest possible momentum change along the trajectory. When compared to the usual least action principle approach, this would correspond to the partial derivative of the action versus time yielding energy and the tendency of the system towards the smallest energy. However, within the least action principle of Maupertuis causality is fundamental in contrast to time, but time can be reconstructed as a dynamical discrete variable yielding a time asymmetric description of mechanics. This approach highlights the fundamental nature of the causal chain of events, and yields the presence of antecedence requested by mechanical causality, which means that an effect cannot occur from a cause that is not in the past, and shows that time is not a fundamental entity.

The definition of causality presented in this paper along with a discrete dynamical time resolves the most important counter arguments against causality in physics, including the time symmetry challenge (denoted challenge (v) above), which is absent in discrete time when actions occur, the vagueness challenge (challenge (i)) because mechanical causality is mathematically defined here, and the determinism challenge (challenge (iii)) because the introduced Maupertuis causality principle is deterministic. Considering the latter point, Poincaré’s citation from the introduction is worth revisiting. Within the least action principle approach, the target of interest (i.e., molecule) indeed appears to know the selection of the most convenient path something like a “living” entity. A possible solution on the request from Poincaré to replace it by a “less objectionable enunciation” is presented here by the reduced action and its defined relationship to causality. As such, the idea presented in this paper re-establishes causality [23].

The concept presented in Section 2.2 appears to be limited to classical physics and is not applicable either to relativity, because time symmetry seems to be requested therein, nor to quantum mechanics, because it lacks causality. However, the Lorentz transformation including the expected invariance can be fulfilled by a discrete dynamical time as shown in reference [22], again using the reduced action as demonstrated above (see Section 2.3), demonstrating the extension of the concept presented to special relativity. In addition, because time in quantum mechanics is of a classical nature [24,25] (since there is no time operator unless quantum mechanics is extended [26]) and with it the time-dependent Schrödinger equation is semiclassical, causality is present after the measurement at the classical level as also highlighted by a causality principle derived as a theorem of quantum theory [23]. This goes in line with the suggestion that time does not exist within quantum mechanics [27]. Within this context it is noted that in standard time-continuous quantum mechanics time asymmetry and an arrow of time is not obtained with the measurement (sometimes called the collapse of the wave function) [28] pinpointing its origin to the classical, macroscopic or/and relativity physics frame, or by introducing stochasticity as done by Gisin with the request on the nonexistence of irrational numbers in nature [29], or in causal set theory [30].

To let time go [31,32,33,34] and replace its function by a causality principle is a difficult task apparently, but to the authors it is a much easier task than to let go of causality. However, further elaborations on the concept of causality both at the mathematical [35,36] and physical level [1,23,37] and the link to the macroscopic arrow of time [18,38,39,40,41,42] are indicated to be key for a further development of fundamental physical theories. With the introduction of causality introduced here, time can only be reconstructed as a discrete entity also yielding other consequences from entropy [16,18,38] to the evolution of the universe [43] and many other findings [44,45,46,47,48], and thus opening more avenues to be studied. We, therefore, invite the reader to participate in this journey. 

## Data Availability

Not applicable.
